# Maternal Sirolimus Therapy for Fetal Extensive Lymphatic Cystic Malformation: Selection Criteria, Management, and Outcomes in a Series of Six Cases

**DOI:** 10.1002/pd.70230

**Published:** 2026-08-17

**Authors:** L. Guibaud, M. Bordas‐Fournel, S. Cabet, C. Le Vaillant, A. Bruel, T. Lefrançois, C. Megier, A. Benachi, M. Sohier, Y. Gallois, T. Simon, J.‐M. Jouannic, G. Canaud, A. Guilhem, A. Atallah, A. Fraissenon

**Affiliations:** ^1^ Pediatric and Fetal Imaging Reference Center for Superficial Vascular Anomalies Hôpital Femme Mère Enfant Université Claude Bernard Lyon 1 Lyon‐Bron France; ^2^ INSERM U1151 Necker Enfants Malades Institute Paris France; ^3^ Multidisciplinary Center for Prenatal Diagnosis Hôpital Femme Mère Enfant Lyon‐Bron France; ^4^ Department of Obstetrics and Gynecology CHU Grenoble Alpes Nantes France; ^5^ Multidisciplinary Center for Prenatal Diagnosis CHU de Nantes Nantes France; ^6^ Service de Néphro‐Pédiatrie CHU de Nantes Nantes France; ^7^ Service d’Imagerie Pédiatrique CHU de Nantes Nantes France; ^8^ Multidisciplinary Center for Prenatal Diagnosis Hôpital Antoine‐Béclère AP‐HP Paris France; ^9^ Multidisciplinary Center for Prenatal Diagnosis Maternité Paule de Viguier CHU de Toulous Toulouse France; ^10^ ORL Pédiatrique Hopital Pierre Paul Riquet CHU de Toulous Toulouse France; ^11^ Service de Néphrologie Pédiatrique Hôpital des Enfants‐Purpan CHU de Toulous Toulouse France; ^12^ Multidisciplinary Center for Prenatal Diagnosis Hôpital Trousseau AP‐HP Paris France

## Abstract

**Objective:**

Maternal sirolimus therapy has emerged as a potential prenatal treatment for extensive fetal lymphatic malformations, particularly when associated with the risk of neonatal airway compromise. Data on prenatal indications, pharmacokinetics, and outcomes remain limited. We aimed to describe patient selection, treatment management, maternal and fetal safety, and early outcomes.

**Methods:**

Between December 2023 and May 2025, 15 fetuses with extensive cystic or mixed vascular anomalies suggestive of lymphatic malformations were referred to a national reference center. Prenatal ultrasound and fetal magnetic resonance imaging were performed. After maternal evaluation and informed consent, sirolimus was administered with weekly dose adjustments based on maternal trough levels. Tolerance, drug concentrations at delivery, obstetric outcomes, and neonatal evolution were assessed. Decrease in lesion extent was assessed using a semi‐quantitative 0–4 imaging scale.

**Results:**

Six fetuses received therapy after the exclusion of nine cases. Treatment began between 24 and over 30 weeks' gestation. Maternal adverse effects were mild. Transplacental drug transfer was observed with variable maternal–fetal ratios. Mean gestational age at birth was 38.1 weeks. Partial or marked lesion regression occurred in five newborns.

**Conclusions:**

Maternal sirolimus therapy appears feasible and well tolerated for extensive fetal lymphatic malformations, demonstrating transplacental transfer and encouraging early perinatal outcomes in carefully selected pregnancies overall.

## Summary

- What's already known about this topic?
  ◦ Maternal sirolimus has been proposed as a prenatal treatment for extensive fetal lymphatic malformations, but evidence is limited to isolated case reports without standardized criteria or management protocols.
- What does this study add?
  ◦ This case series defines reproducible fetal selection criteria and proposes a structured management protocol for maternal sirolimus therapy.
  ◦ It documents materno‐fetal pharmacokinetics and shows frequent volumetric response, particularly when treatment is initiated before the third trimester.

## 1 Introduction

The use of anti‐angiogenic therapies, particularly mechanistic targets of rapamycin inhibitors, has expanded the therapeutic options for complex slow‐flow vascular anomalies and is increasingly considered a first‐line treatment in selected situations [1, 2]. This approach has also been reported in the neonatal period [3]. In a series of 10 neonates with extensive cervicofacial microcystic or mixed lymphatic malformations (LM), we recently confirmed the value of early sirolimus treatment combined with macrocyst sclerotherapy [4], in line with previous neonatal observations reported by Maruani et al. [5].

These postnatal results raised the question of whether even earlier exposure might improve response in severe fetal lesions. In this context, Seront et al. first proposed prenatal maternal sirolimus therapy (MST) and reported successful treatment of a large cervicofacial LM after maternal oral sirolimus initiation at 22 weeks of gestation, with a favorable long‐term postnatal outcome [6]. Since then, prenatal MST for slow‐flow vascular anomalies has been reported only in a very limited number of cases: three case reports described four fetuses treated with MST [7, 8, 9], including one case managed by our group [8], and a short recent series described four additional fetuses treated for different vascular anomalies [10].

Despite these encouraging reports, prenatal MST remains experimental and data on structured treatment protocols remain extremely limited. Major uncertainties persist regarding patient selection, treatment timing, dosing and titration strategies, transplacental pharmacokinetics, maternal and fetal tolerance, and short‐ and long‐term outcomes. At the same time, extensive fetal LM may be associated with severe perinatal morbidity, including airway compromise and complex postnatal management, highlighting an important unmet clinical need.

In this study, we report our experience with six fetuses treated with MST for LM among 15 fetuses referred to our center for discussion of this prenatal approach in the setting of extensive, predominantly cystic, superficial soft‐tissue lesions. Our aim was to describe our selection criteria, treatment management, maternal and fetal tolerance, and prenatal and postnatal outcomes.

## 2 Materials and Methods

### 2.1 Study Design and Referral Pathway

Between December 2023 and May 2025, all consecutive patients referred to our National Reference Center for Superficial Vascular Anomalies for discussion of maternal sirolimus therapy in the setting of a fetal soft‐tissue lesion suggestive of lymphatic malformation were prospectively evaluated as part of routine clinical care. Therefore, this study represents a consecutive single‐center case series conducted in a national referral center.

For patients referred from outside institutions, all available prenatal imaging data, including fetal ultrasound and fetal magnetic resonance imaging, were centrally reviewed. For internally referred patients, prenatal imaging was performed in our Department of Fetal and Pediatric Imaging, which hosts our reference center.

### 2.2 Diagnostic Assessment and Multidisciplinary Evaluation

The diagnostic work‐up was based on detailed fetal ultrasound and fetal MRI to assess lesion extent and severity and to exclude alternative diagnoses, particularly cystic teratoma. Imaging review focused on anatomical extent, deep tissue infiltration, multicompartment involvement, extension to critical anatomical structures, mass effect, and actual or anticipated airway compromise.

Because of the highly infiltrative and multicompartmental nature of extensive lymphatic malformations, with poorly demarcated borders and variable extension across several anatomical spaces, reliable volumetric measurements were not feasible in all cases. In addition, prenatal imaging examinations were performed at different gestational ages and included both ultrasound and fetal MRI, with acquisition planes that were not strictly identical. Under these conditions, formal volumetric assessment would likely have provided a misleading impression of precision and limited reproducibility.

Therefore, lesion evaluation relied on a global radiological assessment integrating maximal lesion extent, depth of infiltration, and mass effect on adjacent anatomical structures. All prenatal imaging examinations were reviewed in consensus by two senior radiologists specialized in fetal imaging and vascular anomalies.

All cases were discussed in a multidisciplinary setting involving specialists in fetal medicine, pediatric radiology, and vascular anomalies. Prenatal MST was considered only after expert review of the imaging findings and assessment of the expected perinatal risk.

### 2.3 Off‐Label Framework, Counseling, and Eligibility for Treatment

MST during pregnancy was proposed as an off‐label treatment with an exclusively fetal therapeutic intent and no expected direct maternal clinical benefit. Parents received detailed counseling regarding the experimental nature of prenatal MST, the limited available prenatal data, the expected benefits, the uncertainties regarding efficacy, and the potential maternal and fetal risks.

The main alternative management option discussed was close prenatal ultrasound surveillance in a national referral center, with perinatal planning and postnatal treatment tailored to lesion evolution and neonatal status. Maternal oral and written informed consent was obtained before treatment initiation in all cases.

Cases were considered eligible for prenatal MST when fetal LM was judged to be extensive and at risk of significant perinatal morbidity. In our center, “extensive” LM was defined using combined anatomical and clinical criteria, including one or more of the following: large anatomical extent, deep tissue infiltration, multicompartment involvement, extension to critical anatomical structures, actual or anticipated airway compromise, or a high expected risk of major perinatal functional impairment.

Cases were not considered for prenatal MST when the lesion appeared limited and compatible with standard postnatal management alone, when another diagnosis was considered more likely on imaging review, when parents declined maternal treatment after counseling, or when fetal evolution no longer supported a meaningful prenatal therapeutic intervention.

## 3 Maternal Pretreatment Assessment

Before treatment initiation, all patients underwent maternal pretreatment assessment, including clinical evaluation, blood pressure measurement, review of potential drug interactions, complete blood count, renal function assessment, liver function tests, lipid profile, and urine dipstick testing.

## 4 Maternal Sirolimus Treatment Protocol

Maternal sirolimus was initiated without a loading dose. The starting dose ranged from 4 to 6 mg once daily according to the clinical situation, followed by maintenance dosing adjusted according to maternal whole‐blood sirolimus trough levels.

This therapeutic strategy was based on the hypothesis that earlier treatment initiation during pregnancy, resulting in longer in utero fetal exposure, might improve treatment response. Given the 30% transplacental sirolimus transfer reported by Seront et al. on cordocentesis‐derived levels, a maternal trough target of 10–12 ng/mL was chosen instead of usual 4–12 ng/mL, to reach a fetal level within the therapeutic range. Dose adjustments were performed in 2‐mg increments or decrements according to measured maternal trough levels in order to reach the target range as early as possible. The maximum dose used in this series was 14 mg per day.

Maternal trough‐level monitoring was performed twice weekly. Maternal blood samples were collected 24 hours after the last sirolimus dose in a non‐fasting state.

For clarity and reproducibility, the complete protocol for maternal sirolimus therapy, including the dosing strategy, pharmacological monitoring, and follow‐up schedule, is provided in Box 1.

**Box 1 Maternal sirolimus therapy protocol for the prenatal management of extensive fetal lymphatic malformations**

1

**Table:** 

DIAGNOSIS	
Diagnostic assessment	Detailed fetal ultrasound and fetal MRI (ideally at 24–26 weeks' gestation) were performed to evaluate lesion extent, severity, deep infiltration, multicompartment involvement, extension to critical anatomical structures, and actual or anticipated airway compromise, and to exclude differential diagnoses, particularly cystic teratoma.
Multidisciplinary evaluation	All cases were reviewed in a multidisciplinary expert center before treatment decision.
Eligibility for prenatal treatment	Prenatal maternal sirolimus therapy was considered only for extensive fetal lymphatic malformations at a high risk of significant perinatal morbidity.
Alternative management	Close prenatal ultrasound surveillance in a national referral center with perinatal planning and postnatal treatment tailored to lesion evolution and neonatal status.
MATERNAL SIROLIMUS THERAPY	
Maternal pretreatment assessment	Clinical evaluation, blood pressure measurement, review of potential drug interactions, complete blood count, serum creatinine, liver function tests, lipid profile, and urine dipstick testing.
Framework and consent	MST was proposed as an off‐label treatment with exclusively fetal therapeutic intent. Maternal oral and written informed consent was obtained in all cases.
Starting dose	Sirolimus was initiated at 4–6 mg once daily according to the clinical situation. No loading dose was used.
Maintenance dose and target exposure	Maintenance dosing was adjusted according to maternal whole‐blood sirolimus trough levels, targeting 10–12 ng/mL.
Dose adjustment	Dose modifications were performed in ± 2 mg increments according to trough levels and maternal tolerance. The maximum dose used in this series was 14 mg/day.
Pharmacological monitoring	Maternal trough levels were measured weekly on blood samples collected 24 hours after the last dose, in a non‐fasting state.
Maternal and fetal follow‐up	Weekly maternal day‐hospital follow‐up included clinical evaluation, blood pressure measurement, complete blood count, renal and liver function tests, lipid profile, and urine dipstick testing. Fetal ultrasound was performed every 2 weeks. Fetal MRI was repeated according to lesion evolution, with at least one examination ≥ 4 weeks after MST initiation.
Treatment continuation or discontinuation	Continuation, dose reduction, temporary interruption, or discontinuation were discussed according to maternal trough levels, maternal tolerance, gestational age, and prenatal lesion evolution. Treatment discontinuation was considered in cases of lesion progression despite therapy or significant maternal toxicity.
Maternal adverse events	Mild to moderate mucositis and hypertriglyceridemia.
DELIVERY	
Delivery planning	Delivery was planned at term whenever feasible, either vaginally or by cesarean section, depending on obstetric and fetal indications, including fetal neck extension.
Treatment continuation	In the absence of major maternal toxicity, sirolimus was continued until the day of delivery.
Assessment of fetal exposure	Umbilical cord blood sampling at delivery was performed to assess fetal sirolimus exposure and to compare cord blood and maternal trough levels.
Management of treatment failure or incomplete response	In cases of treatment failure or incomplete response, postnatal molecular analysis, including assessment for a *PIK3CA*variant when available, was used to guide further management and consider alternative targeted therapy such as alpelisib.

Maternal follow‐up was performed during weekly day‐hospital assessments and included clinical evaluation, blood pressure measurement, fetal heart rate monitoring, complete blood count, renal and liver function tests, lipid profile, and urine dipstick testing. Maternal tolerance and fetal response were assessed weekly.

Fetal follow‐up was based on ultrasound examinations every 2 weeks. Fetal MRI was performed according to lesion evolution, with a minimum of one MRI examination during follow‐up, including one around 27 weeks of gestation, in order to reassess lesion extent, anatomical relationships, and structures at risk.

Treatment continuation, dose reduction, temporary interruption, or discontinuation was discussed according to maternal trough levels, maternal tolerance, gestational age, and prenatal lesion evolution. In our practice, treatment discontinuation was considered in cases of lesion progression on prenatal ultrasound, corresponding to non‐response or worsening despite treatment.

### 4.1 Delivery and Assessment at Birth

Delivery planning aimed at full‐term birth whenever feasible, either vaginally or by cesarean section, depending on obstetric and fetal indications. Sirolimus was continued until the day of delivery in the absence of major maternal toxicity.

At delivery, umbilical cord blood sampling was performed to assess fetal exposure to sirolimus at birth and to compare umbilical cord blood and maternal trough levels.

### 4.2 Outcome Assessment and Postnatal Management

Lesion evolution was assessed by serial prenatal ultrasound and fetal MRI, and again at birth using clinical examination and postnatal imaging. Because formal volumetric measurements were not consistently feasible, lesion response was evaluated using a global radiological impression integrating overall lesion extent, depth of infiltration, and mass effect on adjacent structures.

Response was graded using an ordinal 0–4 scale defined as follows:

0 = no visible change or lesion progression;

1 = mild decrease (estimated < 25% reduction in overall lesion extent);

2 = moderate decrease (approximately 25%–50% reduction);

3 = major decrease (approximately 50%–75% reduction with clear reduction of mass effect);

4 = almost complete regression, with only minimal residual signal or subtle thickening without significant mass effect.

Non‐response was defined as lesion increase during prenatal follow‐up.

Postnatal management was adapted according to lesion persistence and clinical severity, including continuation of sirolimus therapy, sclerotherapy, and, in selected cases, genotyping to document a PIK3CA variant and consider alternative targeted therapies.

## 5 Results

### 5.1 Referred Cases and Eligibility Assessment

Fifteen patients were referred to our national reference center to discuss maternal sirolimus therapy for an extensive fetal soft‐tissue lesion suggestive of lymphatic malformation (LM). Lesion locations included the cervicofacial region (*n* = 12), the hemithorax (*n* = 2, with ipsilateral upper‐limb extension in one case), and the peritoneal cavity with abdominal wall extension (*n* = 1).

**FIGURE 1:**
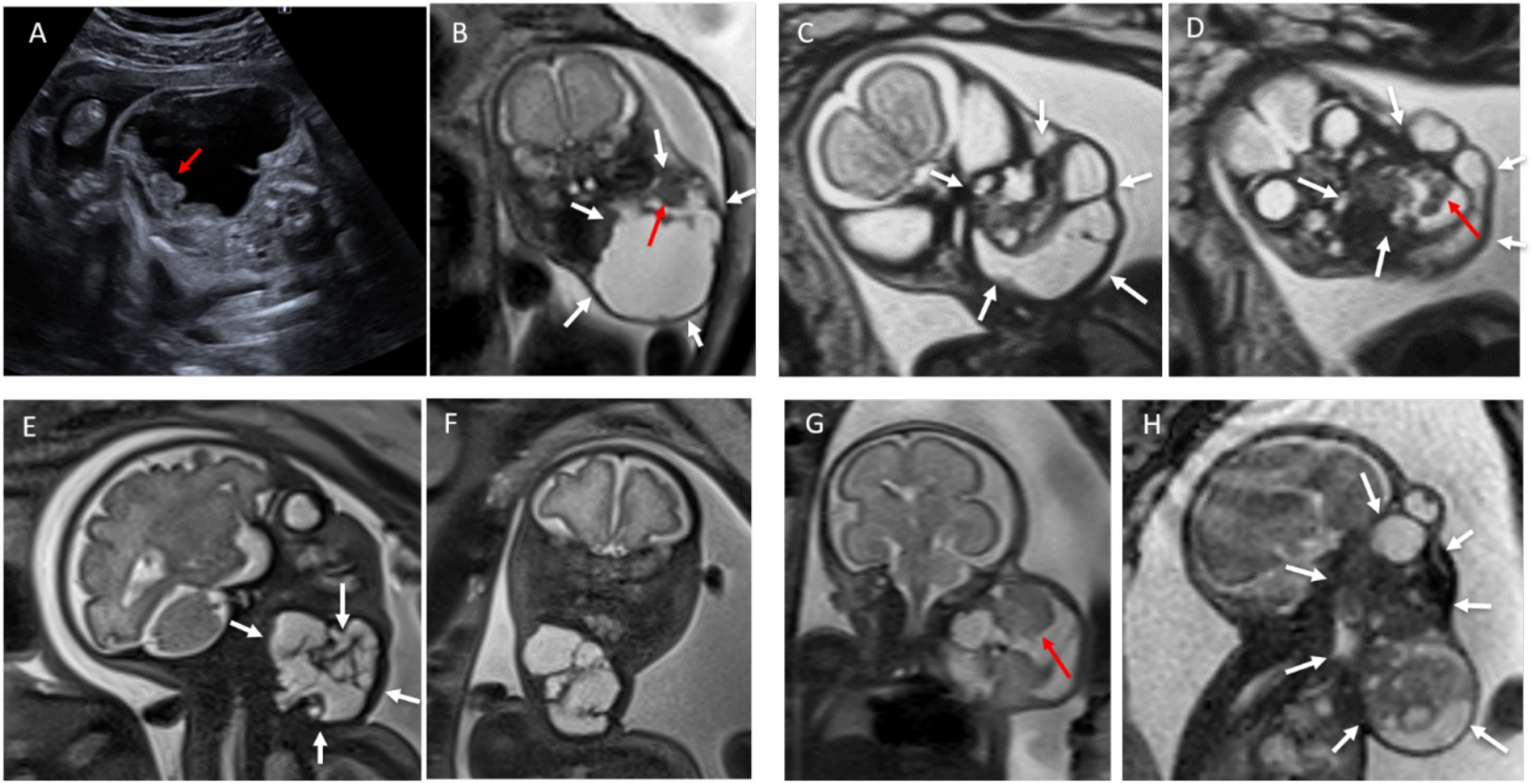
(A‐H): Prenatal imaging of four of our 15 patients referred for extensive cevico‐facial cystic lesion suggestive of LM, in whom review of the ultrasound and fetal MRI examinations raised suspicion of teratoma. Review of the prenatal imaging demonstrated a well circumscribed lesion (small white arrows) with intracystic solid vegetation (red arrow) with major mark effect of this orbite for Patient 4 (H). Patient 1: A,B, Patient 2: C,D, Patient 3: E,F, Patient 4: G,H. In all cases, diagnosis of teratoma was confirmed either pathologically or post‐natally.

**FIGURE 2:**
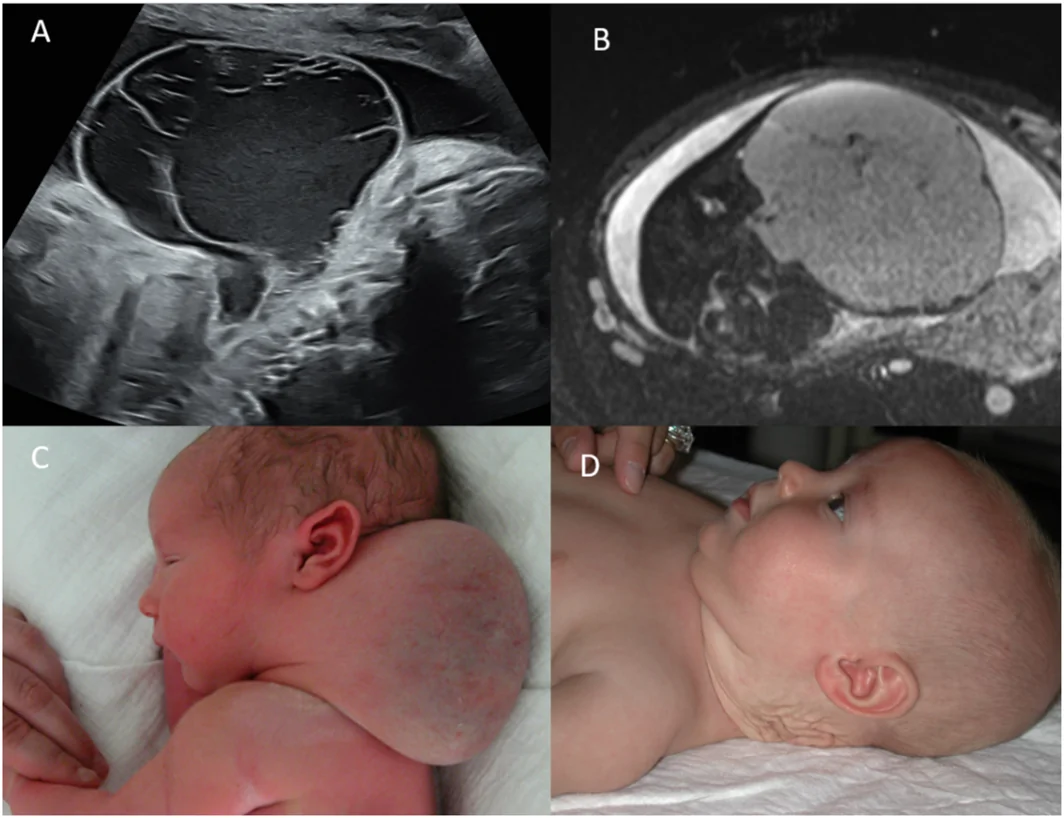
(A‐D): Patient referred for extensive cervico‐facial cystic lymphatic malformation. Prenatal ultrasound at 28 weeks' gestation demonstrated a large cervicofacial cystic lymphatic malformation. Fetal MRI performed at 29 weeks' gestation showed a predominantly pedunculated lesion without deep cervical extension. Based on these imaging findings, maternal sirolimus therapy was not indicated and postnatal sclerotherapy was planned. Postnatal clinical appearance at birth and clinical evolution 4 months after sclerotherapy performed in our referral center showed a favorable outcome.

**FIGURE 3:**
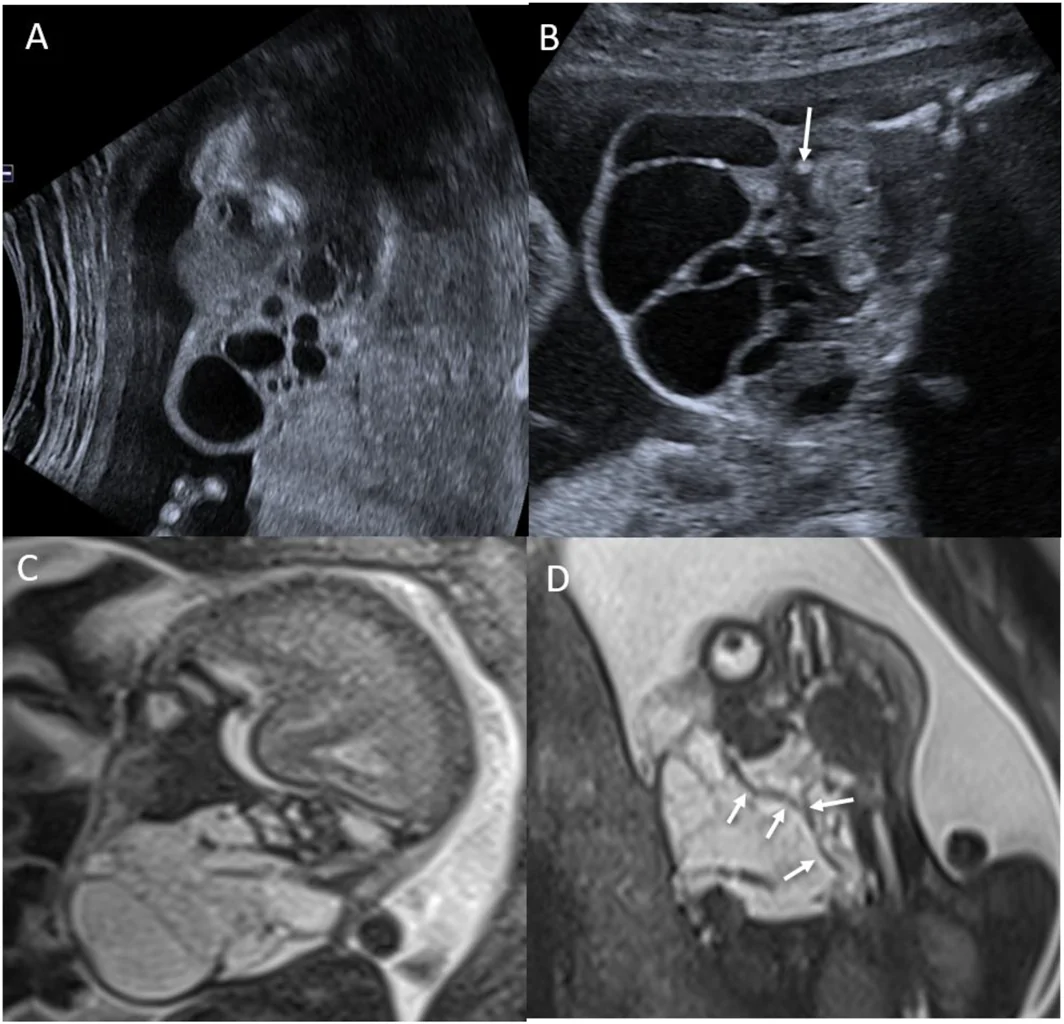
(A–D) The patient referred for extensive cervico‐facial cystic lesion suggestive of LM not included in our series due to fetal death before MST. In this case, sagittal (A) and axial (B) sonographic images performed in the second trimester showed a mainly cystic lesion with a focal echogenic spot on the axial view suggestive of calcification. On these sonographic features, cystic teratoma could not be ruled out. Sagittal (C) and coronal (D) T2‐weighted MR images showed an infiltrative lesion. Note infiltration by the LM around the main carotid artery with clear identification of the division into internal and external carotid arteries without any mass effect. This finding is highly suggestive of LM since such an absence of mass effect on the vessels would not be encountered with a teratoma.

Nine patients were not treated with prenatal MST. Five were excluded after prenatal imaging review because the lesions were considered more suggestive of cystic teratoma and were subsequently confirmed in all cases. Two further cervical lesions were excluded because of limited extent. Representative imaging findings leading to exclusion for suspected teratoma are shown in Figure 1(A‐H), and Figure 2A‐D illustrates a predominantly pedunculated lesion not considered suitable for prenatal MST. In one case, the parents declined treatment and opted for termination of pregnancy, and in another, fetal death occurred before treatment initiation. The referral process, eligibility assessment, exclusions, and final inclusion are summarized in the flow chart. Prenatal imaging findings in the case complicated by fetal death before treatment initiation are shown in Figure 3(A‐D).

### 5.2 Characteristics of Treated Cases

Six patients received prenatal MST. Treatment was initiated between 24 and 28 weeks' gestation in three cases, between 29 and 30 weeks' gestation in two cases, and after 30 weeks' gestation in one case. Baseline characteristics of the treated cases are summarized in Table 1.

**TABLE 1:** Clinical characteristics, therapeutic parameters, and sirolimus levels in six fetuses treated with maternal sirolimus therapy.

	Case 1	Case 2	Case 3	Case 4	Case 5	Case 6
Maternal age	24	33	29	35	30	26
Co‐treatment	/	/	/	/	/	/
Gestational age at MST	33	29	24	27	30	27
Initial dosage (mg)	6	6	6	6	6	6
Maximum dosage (mg)	6	14	6	12	14	12
Decrease of the LM volume	1	2	4	4	3	0
Gestational age of delivery	39.3	39.2	35	37.4	39.5	38.2
Mode of delivery	C	C	V	V	V	C
Birth weight (g)	3270	3280	2250	2720	2900	2840
Maternal residual sirolimus level (μg/L)	5.5	12.8	3.6	16.3	12.2	< 1.2*
Fetal residual sirolimus level (μg/L)	9.8	9.9	3.5	9.6	12.6	1.8
Adverse effects	No	No	No	Yes	No	Yes
Continued sirolimus	Yes	Yes	Yes	No	Yes	Yes
Sclerotherapy	Yes	Yes	No	No	No	Yes

Abbreviations: C = cesarean section; V = vaginal delivery.

### 5.3 Maternal Tolerance and Prenatal Imaging Response

**FIGURE 4:**
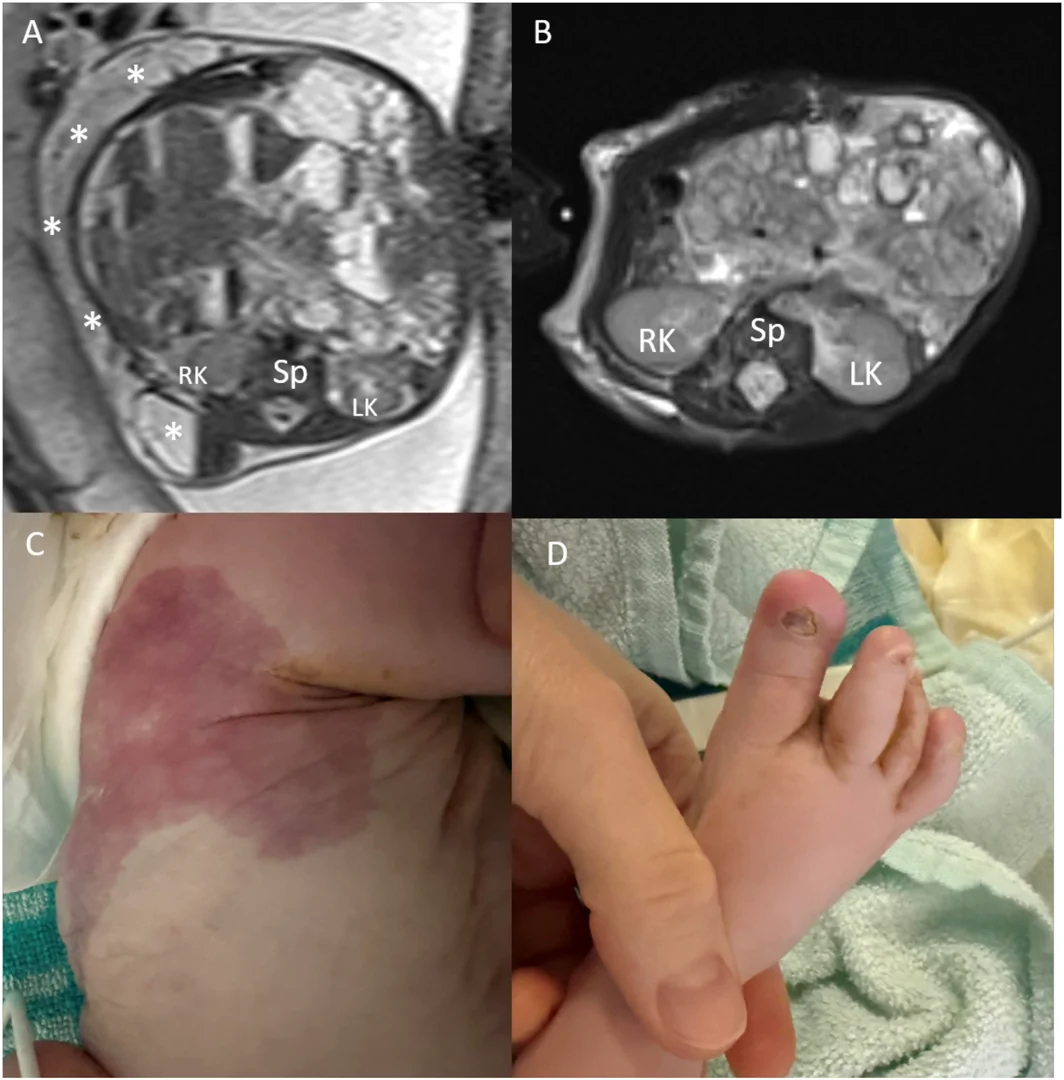
(A‐D): Case 5 with MST at 30 gestational weeks with a good outcome (graded 3). Fetal axial T2 weighted MR image at 28 gestational age at the level of the right and left kidneys (RK, LK) showed an extensive involvement of the whole peritoneal cavity by numerous cystic lesions with fluid‐fluid levels suggestive of internal cystic hemorrhage with diffuse involvement of the right abdominal wall (white *) without clear identification of the intestinal loops (Sp: spine). Post‐natal imaging at the same anatomical level showed a major decrease in both intraperitoneal cysts and abdominal wall infiltration. Neonatal clinical examination showed a large capillary malformation facing the right hemithorax which could not be diagnosed prenatally, which anomalies of the extremities of the left foot, including macrodactyly involving the first and second toes, as well as a sandal gap (excessive distance between the first and second toes), which were overlooked on prenatal imaging.

**FIGURE 5:**
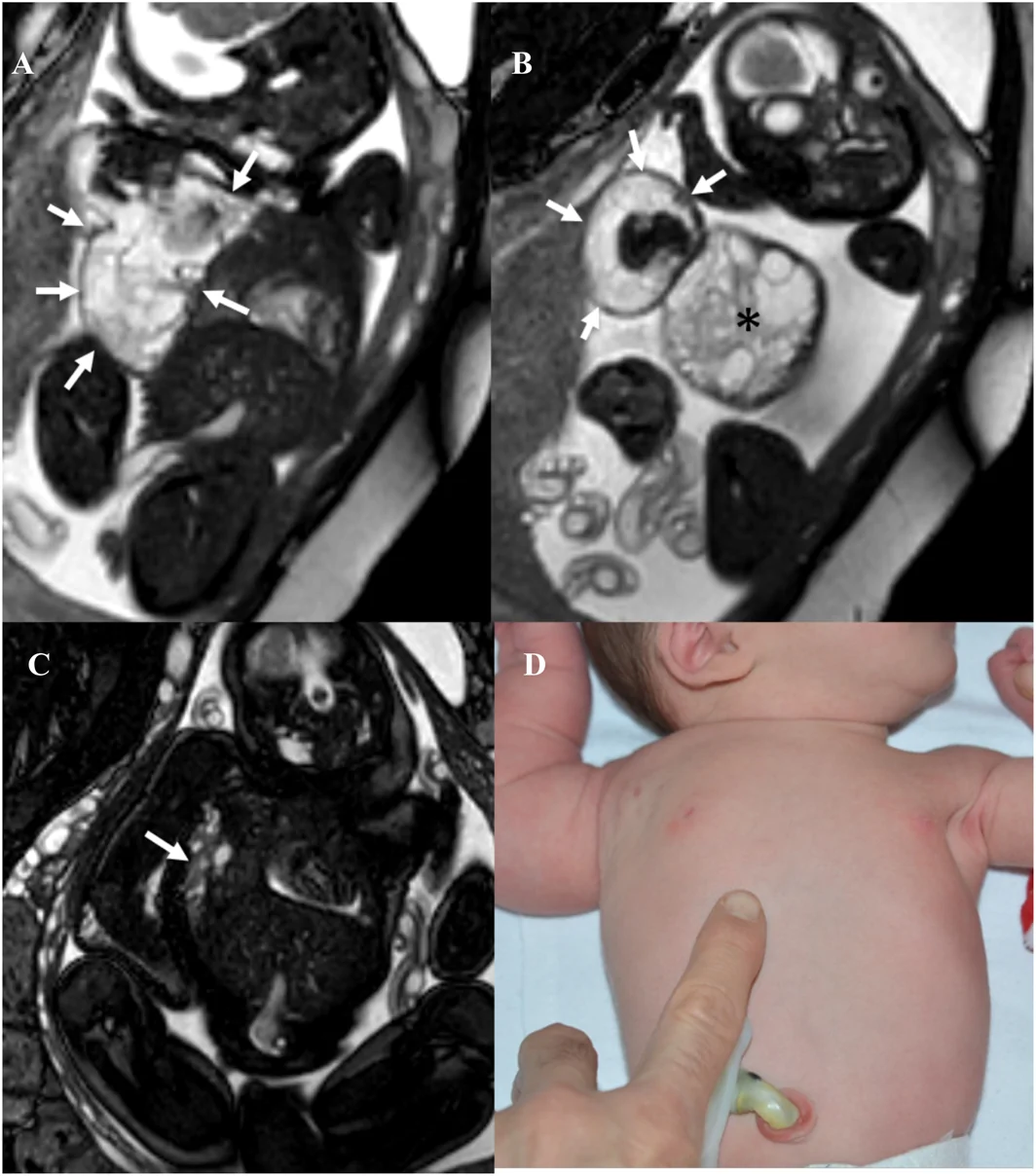
(A–D): Case 4 with MST at 27 gestational weeks with an excellent outcome (graded 4). Coronal T2 weighted coronal MR images (A, B more anterior) showed an extensive LM involving the whole hemithorax with inferior extension facing the right liver (A, white arrows) with a major extension on the anterior thorax (B, black *) and on the right arm (B, white arrows). MR follow‐up at 37 weeks showed a major decrease in the lesion (C, arrow), which was confirmed at birth (D).

**FIGURE 6:**
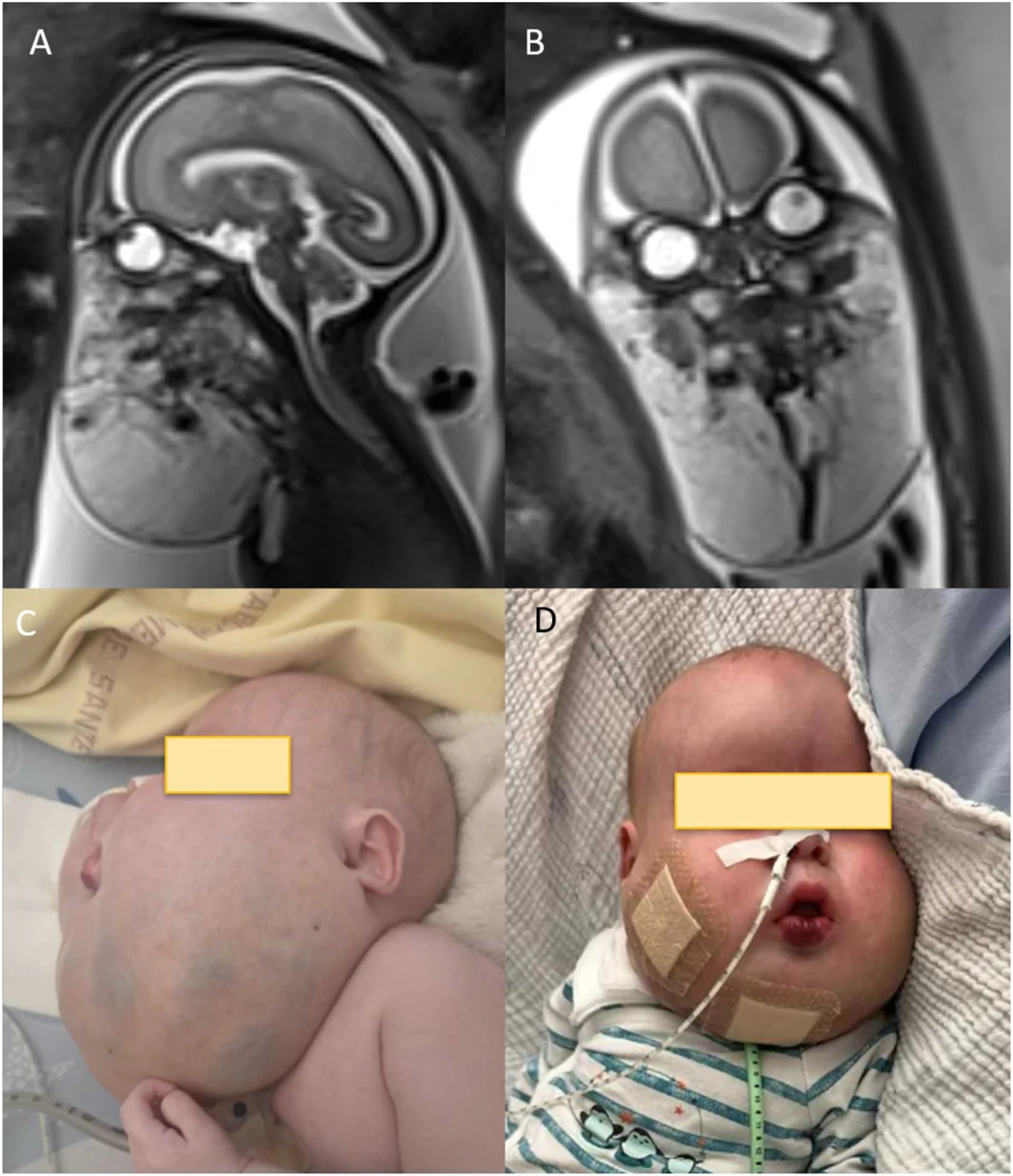
(A‐D): Case with MST at 27 gestational weeks with unexplained absence of response (graded 0). Sagittal and coronal T2 weighted MR images (A, B) showed an extensive LM infiltrating the whole lower face with a major submandibular cystic component extending deeply toward the airways and bilaterally on the parotid areas. Clinical aspect at 3 months without any prenatal and postnatal response to sirolimus, despite additional treatment by PIK3CA targeted therapy (Alpelisib). Clinical aspect at 4 months, 6 weeks after extensive sclerotherapy, showing important decrease in LM. Sirolimus has been discontinued. The baby is still under Alpelisib and dependent on his tracheostomy.

Maternal adverse events were limited to mild or moderate mucositis in two cases (Cases 3 and 6) and hypertriglyceridemia in one case (Case 6). In one case (Case 3), due to the lack of reliable fasting maternal trough levels and limited patient cooperation, the maternal sirolimus dose was reduced to 2 mg/day, corresponding to the standard maintenance dose used in adult transplant recipients. At prenatal imaging follow‐up, lesion decrease was graded as one in Case 1, two in Case 2, three in Case 5, four in Cases 3 and 4, and 0 in Case 6. Representative imaging examples of treatment response are shown in Figures 3, 4, 5, 6.

### 5.4 Delivery and Drug Levels at Birth

Delivery was vaginal in three cases (Cases 3, 4, and 5) and by cesarean section in three cases (Cases 1, 2, and 6). There were one female and five male newborns. Mean birth weight was 2877 g (range, 2250–3280 g), and mean gestational age at birth was 38.1 weeks (range, 35–39.5 weeks). Umbilical cord blood and maternal sirolimus trough levels at delivery were similar in two cases (Cases 3 and 5), higher in cord blood in two cases (Cases 1 and 6), and higher in maternal blood in two cases (Cases 2 and 4). These data are presented in Table 1. The temporal relationship between maternal dosing and maternal trough levels is illustrated in Supporting Information S1: Figure S2.

### 5.5 Neonatal Evaluation and Postnatal Management

At neonatal clinical and imaging evaluations, lesion decrease was graded as one in Case 1, two in Case 2, three in Case 5, four in Cases 3 and 4, and 0 in Case 6. Capillary malformation was identified on dermatological examination in two cases: focal in Case 4 and more extensive in Case 5.

Postnatal sirolimus therapy was continued in all cases except Case 4, in whom the LM had completely disappeared, with no recurrence at 2 years of follow‐up.

Additional sclerotherapy procedures under general anesthesia were performed in three patients: three procedures in Case 1, four in Case 2, and one in Case 6. These procedures allowed tissue sampling for genotyping. A PIK3CA variant was identified in all three tested cases.

In Case 6, no decrease was observed during prenatal follow‐up or during the first 3 months of postnatal sirolimus therapy. This patient subsequently underwent rescue sclerotherapy followed by a treatment switch to alpelisib. In the other five patients, follow‐up showed continued lesion decrease, with near‐complete regression in Case 3.

Overall, prenatal MST was associated with varying degrees of lesion reduction in five of the six treated cases.

## 6 Discussion

### 6.1 Principal Findings

In this single‐center observational series, prenatal maternal sirolimus therapy (MST) was associated with a decrease in lesions in most treated fetuses with extensive lymphatic malformation (LM), with overall acceptable maternal tolerance. Response remained heterogeneous, ranging from almost complete regression to absence of response. Maternal and umbilical cord blood sirolimus trough levels also showed interindividual variability, suggesting that transplacental drug transfer is not uniform across patients.

### 6.2 Comparison With Previous Literature

Prenatal use of mammalian targets of rapamycin inhibitors has been identified as a potential therapeutic option for fetal masses at risk of distress, including cardiac rhabdomyoma and LM [11]. However, published prenatal experiences remain extremely limited. To date, three case reports have described four fetuses successfully treated with MST for LM [7, 8, 9], and Iacobas et al. reported a short series of four fetuses treated for different vascular anomalies, including two with extensive LM [10]. Our cohort therefore adds to a still very limited body of evidence and, to our knowledge, represents one of the largest reported prenatal LM series managed with MST.

Our experience is broadly consistent with previous reports suggesting a potential prenatal benefit of sirolimus in selected severe lesions [6, 7, 8, 9, 10, 11]. At the same time, our data highlight the marked heterogeneity of prenatal presentations, treatment responses, and postnatal trajectories, emphasizing that MST should not be viewed as uniformly effective.

### 6.3 Case Selection and Prenatal Phenotyping

A major practical issue is appropriate case selection. In our experience, careful prenatal phenotyping was essential not only to identify lesions severe enough to justify prenatal treatment but also to avoid misclassification. Several referred fetuses were ultimately excluded because imaging findings were more suggestive of cystic teratoma than LM, underlining that cystic fetal soft‐tissue lesions should not automatically be considered lymphatic malformations [12, 13]. Detailed ultrasound and fetal MRI review are therefore critical to assess lesion extent, infiltrative pattern, associated mass effect, and the presence of solid or vascularized components.

Our data also illustrate the limitations of prenatal phenotyping. Even when prenatal imaging is highly suggestive of LM, more complex vascular overgrowth disorders, including PIK3CA‐related overgrowth spectrum, may only become fully apparent after birth, especially in diffuse microcystic thoraco‐abdominal lesions associated with subtle distal abnormalities or capillary malformations [14]. This uncertainty should be incorporated into prenatal counseling.

### 6.4 Dosing Strategy, Pharmacokinetics, and Transplacental Transfer

Because prenatal MST remains experimental, dosing strategies are not standardized. Our management was initially informed by the experience reported by Seront et al. [6], whose estimated 30% transplacental transfer rate (derived from cordocentesis‐based paired levels) guided our higher maternal trough target (10–12 ng/ml). However our observations suggest that maternal dose, maternal trough level, and fetal exposure are not linked by a simple or predictable relationship. Maternal and umbilical cord blood trough levels at delivery varied substantially between cases, with cord levels being lower, similar to, or higher than maternal levels depending on the patient. These findings do not support a fixed transplacental transfer rate and are consistent with previous suggestions that placental transfer may vary between individuals and across gestation [6, 9, 11].

This variability has important practical implications. This suggests that maternal trough‐level targets may not directly reflect fetal exposure and that empirical dose escalation may not yield equivalent fetal concentrations across patients. More broadly, our data reinforce the need for dedicated pharmacokinetic studies to better characterize maternal‐fetal sirolimus distribution during pregnancy, define appropriate therapeutic targets, and guide standardized dosing strategies [6, 9, 11].

### 6.5 Timing of Treatment and Interpretation of Response

In our series, greater lesion decrease was observed in several fetuses treated during the late second or early third trimester, whereas response appeared more limited in some later‐treated cases. However, given the small number of patients, lesion heterogeneity, and the absence of standardized volumetric assessment, these observations should be interpreted cautiously. Rather than supporting a firm recommendation on optimal timing, our data suggest that treatment timing may influence response and deserves further evaluation in larger cohorts.

The presence of one complete non‐responder despite prenatal treatment also illustrates that resistance or limited sensitivity to sirolimus may occur, for reasons that remain unclear. This further supports the need for better biological and molecular characterization of these lesions.

### 6.6 Clinical Implications

From a clinical perspective, MST should be considered only in highly selected fetuses at high risk of severe perinatal morbidity and within experienced multidisciplinary referral centers. It should be regarded as a potential adjunct to, rather than a replacement for, postnatal management strategies such as sclerotherapy, surgery, and targeted medical therapy. In several patients, postnatal treatment remained necessary despite prenatal exposure.

Our observations also support close maternal, obstetric, fetal, and neonatal monitoring throughout treatment, as well as coordinated prenatal counseling addressing both the potential benefits and the substantial residual uncertainty surrounding this approach.

### 6.7 Strengths and Limitations

The strengths of this study include prospective case evaluation within a national reference center, centralized review of prenatal imaging, explicit treatment selection principles, and combined prenatal and postnatal follow‐up. However, several limitations should be acknowledged. The cohort was small and heterogeneous in terms of lesion location, extent, and gestational age at treatment initiation. The study was single‐center and lacked a control group, preventing any conclusion regarding comparative efficacy. Genetic characterization remained incomplete in some cases, limiting the exploration of genotype‐response correlations.

In addition, response assessment relied on a semi‐quantitative ordinal imaging‐based scale rather than standardized volumetric measurements. However, in the context of extensive lymphatic malformations, which are often highly infiltrative and involve multiple anatomical compartments with poorly defined borders, reliable volumetric assessment is difficult to obtain and may be poorly reproducible. Prenatal imaging was also performed at different gestational ages using both ultrasound and fetal MRI, with acquisition planes that were not strictly identical. Under these conditions, formal volumetric measurements could have provided a misleading impression of precision. For this reason, lesion evolution was assessed using a global radiological evaluation integrating lesion extent, depth of infiltration, and mass effect on adjacent structures, which better reflects real‐life clinical decision‐making in the prenatal management of these complex lesions. Future prospective studies using standardized imaging acquisition, three‐dimensional MRI reconstruction, and dedicated volumetric segmentation tools will be necessary to improve the objectivity and reproducibility of treatment response assessment.

### 6.8 Future Directions

Future work should focus on developing standardized treatment and monitoring protocols, including clearer definitions of eligibility, response, and non‐response. Dedicated pharmacokinetic studies are needed to better understand maternal‐fetal exposure and optimize dosing during pregnancy. Multicenter registries would be particularly valuable to increase sample size, improve phenotypic and genetic characterization, and document both short‐term outcomes and long‐term safety after in utero exposure. Extended follow‐up will be essential to assess growth, neurodevelopment, and other potential long‐term consequences of prenatal sirolimus exposure.

## 7 Conclusion

Maternal sirolimus therapy may represent a potential prenatal treatment option for selected cases of extensive fetal lymphatic malformation, particularly in expert multidisciplinary settings. In our small observational series, prenatal MST was associated with lesion decrease in most treated cases and with an overall acceptable maternal tolerance profile.

Our observations also suggest interindividual variability in transplacental sirolimus transfer, indicating that placental and pharmacokinetic factors may influence fetal drug exposure. These findings support the need for dedicated pharmacokinetic studies to better define target exposure and optimize dosing strategies during pregnancy.

Given the small sample size, the heterogeneity of lesions, and the absence of a control group, our results should be interpreted cautiously. Prenatal MST should therefore remain restricted to highly selected cases at high risk of severe perinatal morbidity, within experienced referral centers, pending further standardized and multicenter evaluation.

These findings may contribute to the development of future standardized protocols for prenatal sirolimus therapy in severe fetal lymphatic malformations.

## Funding

The authors have nothing to report.

## Ethics Statement

The authors have nothing to report.

## Consent

Written informed consent was obtained from all pregnant women as well as from all parents or legal guardians prior to inclusion in the study.

## Conflicts of Interest

The authors declare no conflicts of interest.

## Supporting information


Supporting Information S1

## Data Availability

The data that support the findings of this study are available from the corresponding author upon reasonable request.
