# Supplementary material for: Maternal Sirolimus Therapy for Fetal Extensive Lymphatic Cystic Malformation: Selection Criteria, Management, and Outcomes in a Series of Six Cases
Source: Prenat Diagn. 2026 Aug 17;46(10):1626–36. doi: 10.1002/pd.70230 (PMC13571794; doi:10.1002/pd.70230)
Supplement: Supplementary file 1 [file PD-46-1626-s001.docx]

**Figure S1** Flow chart of patient selection and inclusion for maternal sirolimus therapy

**Figure S2 Temporal relationship between maternal sirolimus dose and maternal whole-blood sirolimus trough levels in the six treated cases.**

Case 1

Case 2

Case 3

Case 4

Case 5

Case 6
